# Effects of cannabinoid exposure on short-term memory and medial orbitofrontal cortex function and chemistry in adolescent female rhesus macaques

**DOI:** 10.3389/fnins.2022.998351

**Published:** 2022-09-30

**Authors:** Stephen J. Kohut, Lei Cao, Dionyssios Mintzopolous, Shan Jiang, Spyros P. Nikas, Alexandros Makriyannis, Chun S. Zou, J. Eric Jensen, Blaise B. Frederick, Jack Bergman, Brian D. Kangas

**Affiliations:** ^1^Department of Psychiatry, Harvard Medical School, Boston, MA, United States; ^2^McLean Imaging Center, McLean Hospital, Belmont, MA, United States; ^3^Behavioral Biology Program, McLean Hospital, Belmont, MA, United States; ^4^Center for Drug Discovery, Northeastern University, Boston, MA, United States

**Keywords:** cannabinoids, delayed match-to-sample task, medial orbitofrontal cortex (mOFC), neuroimaging, non-human primates (NHPs), adolescence

## Abstract

**Aim:**

There is increasing concern that cannabinoid exposure during adolescence may disturb brain maturation and produce long-term cognitive deficits. However, studies in human subjects have provided limited evidence for such causality. The present study utilized behavioral and neuroimaging endpoints in female non-human primates to examine the effects of acute and chronic exposure during adolescence to the cannabinoid receptor full agonist, AM2389, on cognitive processing and brain function and chemistry.

**Materials and methods:**

Adolescent female rhesus macaques were trained on a titrating-delay matching-to-sample (TDMTS) touchscreen task that assays working memory. TDMTS performance was assessed before and during chronic exposure to AM2389, following antagonist (rimonabant) administration, and after discontinuation of the chronic regimen. Resting-state fMRI connectivity and magnetic resonance spectroscopy data were acquired prior to drug treatment, during chronic exposure, and following its discontinuation. Voxels were placed in the medial orbitofrontal cortex (mOFC), a region involved in memory processing that undergoes maturation during adolescence.

**Results:**

TDMTS performance was dose-dependently disrupted by acute AM2389; however, chronic treatment resulted in tolerance to these effects. TDMTS performance also was disrupted by discontinuation of the chronic regimen but surprisingly, not by rimonabant administration during chronic AM2389 treatment. mOFC *N-*acetylaspartate/creatine ratio decreased after acute and chronic administration but returned to baseline values following discontinuation of chronic treatment. Finally, intra-network functional connectivity (mOFC) increased during the chronic regimen and returned to baseline values following its discontinuation.

**Conclusion:**

Neural effects of a cannabinergic drug may persist during chronic exposure, notwithstanding the development of tolerance to behavioral effects. However, such effects dissipate upon discontinuation, reflecting the restorative capacity of affected brain processes.

## Introduction

The use of cannabis and other cannabinergic drugs during adolescence, a developmental period when the brain undergoes maturation, is widespread and continues to grow (Bava et al., 2010; Peters et al., 2022). It is uncertain whether cannabinoid exposure during this time may lead to long-term alterations in cognition and/or brain function (Becker and Hu, 2008); however, a number of studies suggest that repeated exposure can adversely affect cognitive performance (Egerton et al., 2006; Crean et al., 2011; Zanettini et al., 2011). Of special concern, such deleterious effects on cognitive performance have been noted in a range of tasks involving fronto-cortical brain regions, which undergo extensive maturation throughout adolescence (Delevich et al., 2021). For example, deficits in the performance of cannabis users relative to age-matched controls have been observed in tasks of inhibitory control and cognitive flexibility, sustained attention, impulsivity, and memory (Gruber and Yurgelun-Todd, 2005; Fontes et al., 2011; Hanson et al., 2011; Tait et al., 2011; Wrege et al., 2014). Thus, considerable evidence supports the idea that cannabis use during adolescence can have a persistent negative impact on cognitive function.

Disruptions in cognitive performance, especially those related to memory function, previously have been observed in preclinical studies of Δ^9^-tetrahydrocannabinol (Δ^9^-THC) and other cannabinergic drugs in rodents and non-human primates. For example, daily cannabinoid exposure was shown to impair novel object recognition, short-term memory, and cognitive flexibility in adolescent rats (O’Shea et al., 2004, 2006; Cha et al., 2007; Quinn et al., 2008; Higuera-Matas et al., 2009; Zamberletti et al., 2014). Similarly, spatial memory in adolescent monkeys also was disrupted by daily Δ^9^-THC exposure over the course of 6 months (Verrico et al., 2014).

Neuroimaging studies in human subjects have provided insight into the brain regions that underlie different types of task performance (Huettel, 2012) as well as those that may be altered in adolescent cannabis users (Sager and Gruber, 2019). For example, studies of resting-state functional connectivity (RSFC) suggest a relationship between extent of cannabis use and abnormal connectivity within fronto-temporal networks (Houck et al., 2013; Cheng et al., 2014; Camchong et al., 2017). Adolescent cannabis use, in particular, has been associated with reduced interhemispheric RSFC and elevated right hemisphere connectivity that may underlie the disruption of coherent, organized cognitive and behavioral processes (Orr et al., 2013). Adolescent-onset cannabis users also may display altered patterns of brain activity (primarily in fronto-cortical regions) when it is measured during cognitive tasks, including short-term memory of several types (Padula et al., 2007; Tapert et al., 2007; Schweinsburg et al., 2008; Jacobus et al., 2009; Sagar et al., 2015). Although the results of fMRI experiments may vary depending on the particular methodology and regions of interest, the altered pattern of communication between brain areas at rest in cannabis users suggests that qualitative changes in intrinsic connectivity may be related to deficits in cognitive performance.

Research in human subjects to solidify our understanding of the consequences of adolescent exposure to cannabinoids on behavior and brain function faces considerable challenges. For example, due to ethical and practical constraints, such research (especially in adolescents) is predominantly cross-sectional rather than longitudinal. Moreover, it is difficult to control for histories that include the use or abuse of multiple substances as well as for other factors such as duration of use, age of initiation, frequency/amount of consumption, drugged versus withdrawal state (Churchwell et al., 2010; Lorenzetti et al., 2016), and polysubstance use (Moss et al., 2014). From this perspective, the extent to which results in human subjects reflect the effects of cannabis directly on behavior and brain function or, alternatively, contamination by such uncontrolled factors remains uncertain (Cheetham et al., 2012; Lubman et al., 2015). One approach to mitigating the problem of uncontrolled historical or contextual factors in preclinical research is through translational research in laboratory animals and, in particular, non-human primates. The neurological, phylogenetic, pharmacokinetic, and pharmacodynamic aspects of drug action in rhesus monkeys, for example, greatly overlap with those of humans (Weerts et al., 2007), permitting translationally-relevant longitudinal studies of the effects of cannabinoid exposure on complex behavior and brain function during adolescence.

In the present research, a touchscreen-based titrating-delay matching-to-sample task of short-term memory function (TDMTS; Kangas et al., 2010) was paired with longitudinal neuroimaging (fMRI and magnetic resonance spectroscopy) measures to investigate the effects of acute and chronic exposure to the cannabinoid receptor type 1 (CB_1_) agonist AM2389 in adolescent female rhesus macaques. Female macaques were studied in the present work because (1) cannabinergic sex differences have been documented, including a greater CB_1_ density across several brain regions in male than female laboratory animals (e.g., Rubino et al., 2008; Reich et al., 2009; Burston et al., 2010; Mateos et al., 2011), (2) the role of density in regions of interest related to memorial processes, including the orbitofrontal cortex (Lamar et al., 2004), are unknown, and (3) there continues to be a need to address the well-publicized underrepresentation of female subjects in biomedical research (Woitowich et al., 2020). Data also were obtained following chronic treatment to examine the immediate and longer-term effects of discontinuing daily injections of AM2389. Lastly, the effects of the CB_1_ antagonist rimonabant were determined during chronic AM2389 to assess the presence of behavioral or neural signs of antagonist-precipitated withdrawal. AM2389 was selected for study because its long duration of behavioral action (∼17 h *in vivo* half-life; Järbe et al., 2012) assures continuous CB_1_ receptor occupancy with once-daily treatment. In addition, as a CB_1_ receptor full agonist, its agonist effects may be more definitive than those of partial agonists like Δ^9^-THC (Paronis et al., 2012; Kangas et al., 2020). TDMTS task performance was selected as the cognitive endpoint for the present studies in view of the well-known ability of cannabinoids to disrupt short-term memory, an adverse effect that has been repeatedly documented in chronic cannabis users (Crean et al., 2011; Gruber et al., 2012; Crane et al., 2013) and preclinical studies in rodents and non-human primates (e.g., Cadoni et al., 2001; Verrico et al., 2012; Kangas et al., 2016). Neuroimaging procedures have focused on the medial orbitofrontal cortex, which has been shown to be involved in DMTS performance in an age-dependent manner (Lamar et al., 2004) and is also thought to play a critical role in substance abuse (London et al., 2000; Winstanley, 2007; Schoenbaum and Shaham, 2008).

## Materials and methods

### Subjects

Four experimentally- and drug-naïve adolescent female rhesus macaques (*Macaca mulatta*) were studied in the present research. The subjects, which were 3.5–4.0 years old when studies were initiated, were singly housed in a temperature- and humidity-controlled vivarium with a 12-h light/dark cycle (lights on at 7 am). Each subject had continuous access to water in the home cage and was maintained at its approximate free-feeding weight by post-session feedings of a nutritionally balanced diet of fresh fruit, vegetables, and high protein biscuits (Purina Mills International, Brentwood, MO, United States); environmental enrichment (e.g., toys, puzzles, mirrors) was provided daily. The protocol for these studies was approved by the Institutional Animal Care and Use Committee at McLean Hospital and was conducted in a facility licensed by the United States Department of Agriculture and in accordance with guidelines provided by the Committee on Care and Use of Laboratory Animals of the Institute of Laboratory Animals Resources, Commission on Life Sciences (National Research Council, 2010).

### Apparatus

Details and schematics of the touchscreen chamber designed for rhesus macaques can be found in Kangas and Bergman (2017). Subjects were seated in non-human primate chairs (515-SASR, PLAS Labs, Inc., Lansing, MI, United States) within the chamber. Each chair was outfitted with a stainless-steel sipper tube that was readily accessible to the subject’s mouth for the delivery of liquid reinforcement [0.15 ml of 30% sweetened condensed milk dispensed *via* an infusion pump situated outside the experimental chamber (PHM-100-10, Med Associates, St. Albans, VT, United States)]. A 17” touch-sensitive screen (1739L, ELO TouchSystems, Menlo Park, CA, United States) was mounted to an inside wall of the enclosure directly in front of the subject and within easy reach. A speaker bar (NQ576AT, Hewlett-Packard, Palo Alto, CA, United States) was mounted above the touchscreen to provide audible feedback. All experimental events and data collection were programmed in E-Prime Professional 2.0 (Psychology Software Tools, Inc., Sharpsburg, PA, United States).

### Experimental timeline

**FIGURE 1:**
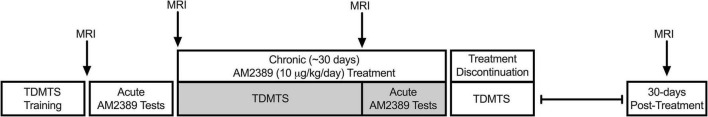
Experimental timeline.

An experimental timeline of behavioral, drug administration, and imaging conditions is presented in Figure 1. As shown, dose-response data for the acute effects of AM2389 on short-term memory were obtained before and after chronic exposure to AM2389, with neuroimaging sessions to obtain MRS and fMRI data at several key time points. Scan sessions prior to initial dose-response determination of the behavioral effects of AM2389 provided baseline control data whereas scan sessions conducted on the first and last days of the chronic regimen provided data before and after the development of tolerance to the effects of AM2389 on short-term memory. Data obtained 30 days following discontinuation of the chronic treatment regimen offered insight into the persistence of neural changes observed during the chronic regimen.

### Titrating-delay matching-to-sample

#### Training procedures

Subjects were first trained to respond on (i.e., touch) the touchscreen using previously described shaping procedures (Kangas and Bergman, 2012). After reliable responding was observed, subjects were next trained on a touchscreen-based matching-to-sample procedure (Kangas et al., 2016). Trials began with presentation of a 7 × 7 cm^2^ pictorial stimulus (either a photograph of green leaves or red cherries) presented in the center of the screen (sample stimulus). After completion of a fixed ratio (FR) 20 response requirement on the sample stimulus, the sample stimulus was replaced by presentation of both stimuli (comparison stimuli) positioned to the left and right of center. The position of the two stimuli—left or right—was assigned randomly from trial to trial. A single response on the comparison stimulus that matched the previously presented sample stimulus initiated the delivery of liquid reinforcement accompanied by an 880 ms yellow screen flash and followed by a 10 s intertrial interval (ITI) blackout. A response on the non-matching stimulus immediately initiated a 20 s ITI blackout. A correction procedure (Kangas and Branch, 2008) was implemented such that incorrect trials were repeated until a correct response was emitted. Sessions ended after the subject completed 48 correct trials. After a subject committed 5 or fewer errors in each of 5 consecutive sessions, it was exposed to the titrating-delay matching-to-sample (TDMTS) task. In this procedure, the contingencies remained the same as described above, with the exception that every 2 consecutive correct matches increased the delay value (retention interval) between sample stimulus offset and comparison stimuli onset by 1-s on the next trial; every incorrect response decreased the delay by 1 s on the next trial (Kangas et al., 2010). Each TDMTS session began with a 0 s delay and training sessions continued until session-wide average titrated delay values were ± 20% of the 5-session mean. Thereafter, drug testing began under identical conditions except that the session was terminated if 30 min elapsed without a response or after 2-hr total, whichever came first. Following the achievement of stable levels of responding per the above criteria, baseline MRI scans were conducted as described below.

### Drug testing and dosing procedures

#### Acute studies

The acute effects of AM2389 on short-term memory performance were determined by injecting vehicle or each of a range of doses (0.56–10.0 μg/kg) of AM2389 intramuscularly (i.m.) 3-hr prior to the TDMTS session. Doses were administered no more than once per week and were studied in a mixed order across subjects. After the completion of dose-response determinations with AM2389, task performance also was assessed 1 h following i.m. administration of the CB_1_ receptor antagonist rimonabant (1.0 mg/kg). These effects of rimonabant in untreated subjects provided control data for evaluating the effects of rimonabant administered during chronic AM2389 treatment.

#### Chronic treatment

After acute studies were completed, subjects were treated once daily in the morning (7 days/week) with AM2389. On the first day of the chronic regimen, subjects received 10 μg/kg, i.m. AM2389 3 h prior to a scanning session. This provided information regarding the acute MRS- and fMRI-related effects of the dose of AM2389 that substantially disrupted task performance in all subjects in preceding dose-response determinations and that was scheduled to be the terminal daily treatment dose in the ensuing chronic regimen. Starting the next day, TDMTS sessions were conducted 3 h after the injection, 5 days/week (Mon-Fri). The treatment dose of AM2389 began at 0.32 μg/kg/day and, in each subject, increased in 0.5 log unit steps after 2 consecutive TDMTS sessions were completed successfully, i.e., 0.32, 1.0, 3.2, 10.0 μg/kg/day. Each subject was scanned after the accumulated chronic dosage of AM2389 reached 0.32 mg/kg, i.e., 39–56 days after the chronic regimen began. Thereafter, to assess the extent of tolerance to the effects of the cannabinoid on TDMTS task performance, the effects of larger AM2389 doses (18.0–56.0 μg/kg) on TDMTS performance were determined in each subject. Larger doses of AM2389 were tested no more than once per week, with the 10.0 μg/kg chronic dose administered on intervening days.

#### Antagonist-precipitated withdrawal and abrupt discontinuation of AM2389

Two approaches were used to determine whether chronic AM2389 exposure produced physical dependence, as revealed by signs of withdrawal, after tolerance to its disruptive behavioral effects was evident. First, experiments were conducted to determine whether TDMTS task performance during the chronic AM2389 regimen was altered by administration of the CB_1_ antagonist rimonabant, presumably as a result of displacing AM2389 from CB_1_ receptors (i.e., antagonist-precipitated withdrawal). In these experiments, the effects of several doses of rimonabant (0.1–1.0 mg/kg) were determined by administering the antagonist 1-hr prior to the test session (i.e., 2-hr after daily treatment with 10.0 μg/kg AM2389). Rimonabant was studied in individual subjects no more than once per week. After tests with rimonabant concluded, chronic treatment with AM2389 continued for 10 days, after which vehicle solution replaced AM2389 as the daily treatment in all subjects. TDMTS performance was evaluated daily during the first 10 days following discontinuation, providing a second measure of withdrawal signs consequent to chronic AM2389 exposure. Finally, subjects were re-scanned 30 days after daily treatment with AM2389 was discontinued to evaluate the persistence of MRS- and MRI-related changes that may have occurred during the chronic regimen.

### Magnetic resonance imaging and spectroscopy (MRI and MRS)

MRI data were acquired on a Siemens Magnetom 3T Trio (Tim syngo MR B17). A custom 5-channel phased-array head coil that was designed to optimize magnetic field homogeneity for rhesus macaque brains was used. On scan days, subjects were transported from the vivarium to an animal preparation room within the scanner suite. Subjects were sedated with 10 mg/kg ketamine i.m. for anesthesia induction, intubated, and then maintained on 1–1.2% isoflurane gas throughout the scanning procedures. A circulating warm-water blanket and fleece wrap were used to maintain body temperature. Monkeys were scanned in the prone position in an MRI-compatible primate holder. Vital signs including heart rate, respiration rate, and oxygen saturation (SPO2) were monitored throughout the procedure.

The image acquisition protocol included (1) a *3D MP-RAGE* high resolution anatomic sequence (non-selective inversion-recovery time TI = 1,100 ms, TR = 2,530 ms, four echoes at TE = 3.69 ms; 7.64 ms; 9.72 ms; 11.8 ms, 128 sagittal slices, slice thickness = 1.3 mm, voxel size = 0.7 × 0.5 × 1.3 mm, FOV = 128 mm, flip angle = 70°, GRAPPA acceleration factor 2, acquisition time = 4 min 32 s. Anatomic images were then used to place a single 7 mm × 7 mm × 11 mm MRS voxel in the right mOFC – targeting an area encompassing 10mc, 10mr, 14c, 14r according to the Saleem and Logothetis macaque atlas (Saleem and Logothetis, 2012; see Figure 5A). Magnetic resonance spectra were next acquired using a *J-resolved 2D-JPRESS* sequence (Jensen et al., 2009; see also Kohut and Kaufman, 2021). Following manual shimming and optimization of the flip angles for water suppression and MRS acquisition, a TE-series comprised of 22 steps was acquired with initial TE = 35 ms, final TE = 350 ms, TE step = 15 ms (66.7 Hz J-resolved bandwidth). The mean TE of the TE-series = 192.5 ms matches the average TE of *in vivo* metabolite T2 values reported by Posse et al. (2007) for a field strength of 3T. Other acquisition details: TR = 2,190 ms, 8 averages per TE step, 2 kHz spectral bandwidth, 1,024 complex pairs per trace, 6:39 min total acquisition time. After acquisition of MRS data, a 10 mg/kg IV injection of Feraheme^®^ (ferumoxytol) was administered and 5-min later, functional MRI data was acquired using a *2D Gradient-echo* EPI sequence (TR = 1,750 ms, TE = 21 ms, 29 slices, slice thickness = 1.8 mm, interleaved, voxel size = 1.4 × 1.4 × 1.8 mm^3^, FOV = 130 mm, flip angle = 70°, GRAPPA acceleration factor 2, 344 volumes, acquisition time = 10 min 7 s).

**FIGURE 2:**
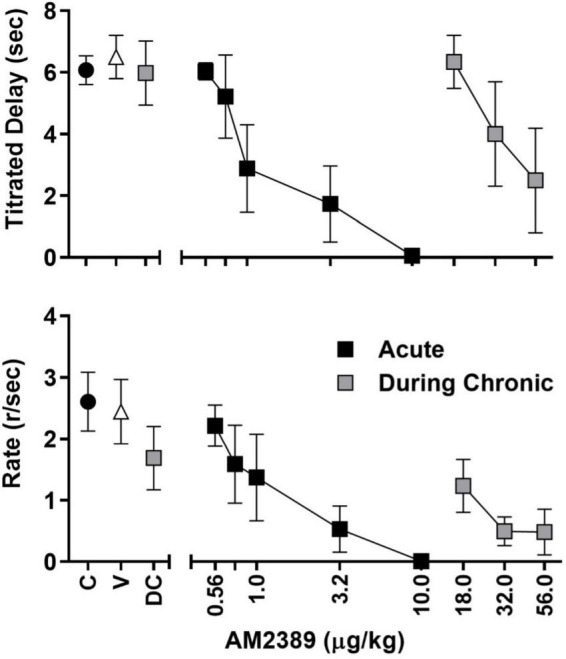
Session-wide mean (± SEM) titrated duration values **(upper panel)** and mean (± SEM) response rate values **(lower panel)** in the TDMTS task during control sessions prior to drug exposure (C; black circles), following vehicle administration (V), during acute dose-response determinations (black squares), during chronic dose-response determinations (gray squares) *N* = 4.

**FIGURE 3:**
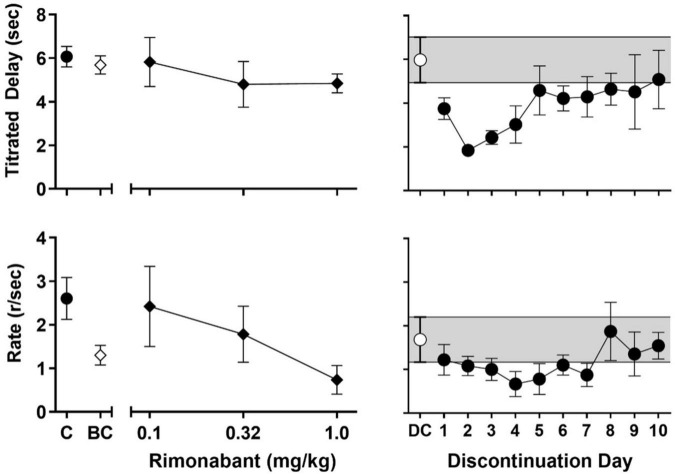
**Left panels:** Effects of rimonabant administration on session-wide mean (± SEM) titrated duration values (upper-left panel) and mean (± SEM) response rate values (lower-left panel) in the TDMTS task before chronic exposure (BC; 1 mg/kg) and in dose-response determinations during chronic treatment (black diamonds; 0.1–1.0 mg/kg). For ease of comparison, values obtained during control sessions prior to drug exposure (C; black circles) are also shown. **Right panels:** Session-wide mean (± SEM) titrated duration values (upper-right panel) and mean (± SEM) response rate values (lower-right panel) in the TDMTS task during the last 5 sessions of chronic AM2389 exposure (DC; while circle) and during the 10 days following abrupt AM2389 discontinuation (black circles) *N* = 4.

**FIGURE 4:**
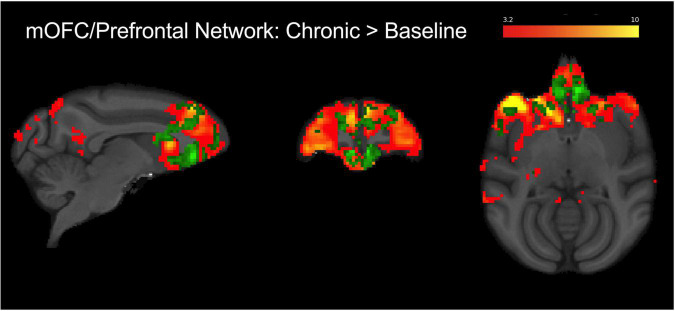
Group level (*N* = 4) fronto-cortical resting-state network spatial maps encompassing the mOFC, prefrontal cortex, and anterior cingulate (red-yellow). Green overlay shows regions identified using dual regression with greater connectivity during chronic AM2389 treatment relative to baseline (*p* < 0.05). There were no statistically significant differences between acute drug scan or discontinuation and baseline. See text for additional details.

**FIGURE 5:**
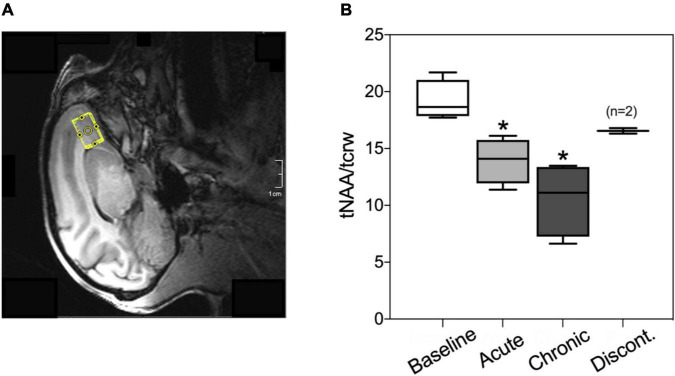
**(A)** Representative voxel placement for spectroscopy measurement at medial orbitofrontal cortex (mOFC) in rhesus macaques. **(B)** Boxplots of *N-*acetylaspartate/creatine ratio (tNAA/tCrw) during baseline, acute administration of AM2389 (10 μg/kg), during chronic AM2389 treatment (total dose 0.32 mg/kg), and 30 days after treatment discontinuation. *p < 0.05 vs. Baseline (*N* = 4).

### Data analysis

#### Titrating-delay match-to-sample

Overall mean titrated duration values were calculated by averaging (± SEM) the titrated duration values across the 48-trial session. Response rate (responses/sec) during presentation of the sample stimulus was calculated by dividing the number of FR responses (20) by the time between the onset and offset of the sample stimulus; overall mean response rate for the session was calculated by averaging values (± SEM) across the number of completed trials. ED_50_ values were determined for both performance (average titrated delay) and response rate using log-linear interpolation in individual subject dose-response functions. Group ED_50_ values (with 95% confidence limits) were determined for comparisons of effects of acute and chronic AM2389 on performance and response rate. Log ED_50_ values were transformed to linear values for presentation.

#### Magnetic resonance spectroscopy

Spectra were processed as single spectra using TE-averaging (Hurd et al., 2004; Jensen et al., 2009). The TE-series were Fourier-transformed to frequency space using jMRUI v4^1^, lightly apodized with a 2 Hz Lorentzian-Gaussian filter and summed. The resultant sum was fitted with LCModel using a dedicated simulated basis within the GAMMA/VESPA simulation software (Soher et al., 2007). The spectra were inspected visually in jMRUI and then fitted with the LCModel to quantify metabolites for spectra with water linewidths less than 10 Hz. The data were analyzed with a mixed-model repeated-measures ANOVA followed by Dunnet’s *post hoc* tests when appropriate. The tests were run for the ratios NAA/tCr and tNAA/tCr. tNAA was estimated by adding NAA and NAAG *via* a standard weighted-sum formula in error analysis (Bevington et al., 1993; Mintzopoulos et al., 2016). All statistical analyses were conducted using Stata (v14; StataCorp, College Station, Texas). Data from two subjects at the discontinuation timepoint were removed due to poor fits.

#### Functional magnetic resonance imaging

Image preprocessing included removing the first seven volumes, motion correction, spatial smoothing with a Gaussian Kernel of 2.5 mm FWHM, high pass temporal filtering using a 100 s filter, and slice timing correction conducted within the FSL software suite (FMRIB, Oxford University, United Kingdom). Processed data were then skull-stripped and registered to INIA macaque template space (Rohlfing et al., 2012) using the JIP software toolkit (Joe’s Image Program^2^, Massachusetts General Hospital, Harvard University, MA, United States). Group independent component analysis (ICA) was conducted to delineate resting state networks that overlapped the right mOFC voxel used in MRS and verified using spatial cross-correlation. Dual regression analysis was used to regress the group spatial maps into each subject’s 4D dataset to give a time course which was then regressed into the same 4D dataset for a subject-specific set of spatial maps (Smith et al., 2009). Permutation testing using the FSL randomize tool was used to compare differences across each condition. Family-wise error (FEW) correction with threshold-free cluster enhancement (TFCE) with a significance threshold of *p* < 0.05 was used to correct for multiple comparisons (Smith and Nichols, 2009).

### Drugs

AM2389 was synthesized for these studies by the present authors (SJ, SPN, AM) in the Center for Drug Discovery at Northeastern University (Boston, MA, United States) using previously published procedures (Nikas et al., 2010). Rimonabant was provided by the NIDA Drug Supply Program (Rockville, MD, United States). AM2389 and rimonabant were prepared in a 20:20:60 mixture of 95% ethanol, Tween-80, and saline. All drug solutions were refrigerated and protected from light. Vehicle and drug doses were prepared in volumes of 0.5 ml/kg body weight or less and administered i.m. in calf or thigh muscle.

## Results

### Titrating-delay match-to-sample

All subjects learned to touch the screen and progressed through matching-to-sample training to stable performance within approximately 60–75 training sessions. Group mean values for titrated delay (Figure 2, upper panel) and rates of responding during the FR20 sample response requirement (Figure 2, lower panel) are depicted by black circles. As shown, session-wide titrated delays averaged approximately 6 s and the FR20 requirement was completed, on average, at the rate of approximately 2.6 responses per sec. AM2389, given acutely, produced dose-dependent decreases in both measures and the highest acute dose, 10.0 μg/kg, abolished responding in all subjects (black squares). Following acute dose-response determinations, subjects were chronically exposed to AM2389 *via* a dosing regimen that, as described above, increased in a graded manner to the terminal daily dose of 10.0 μg/kg AM2389. Mean titrated delay values and FR20 response rates decreased initially but gradually recovered over the course of chronic treatment, presumably reflecting tolerance to the behaviorally disruptive effects of AM2389. Dose-response functions were redetermined when the total AM2389 dosage administered during the chronic regimen reached 0.32 mg/kg which, depending on the subject’s progression through dose escalation, took 39–56 days of once-daily injection. As depicted by the gray squares in the left panels of Figure 2, mean titrated delay values at the time of dose-response re-determination (DC) approximated pre-chronic control values whereas the mean rate of FR20 responding was approximately 70% of pre-chronic response rates. Administration of higher AM2389 doses (18.0–56.0 μg/kg) revealed a > 30-fold rightward shift in the position of the dose-response function [ED_50_ (CLs); acute = 1.68 μg/kg (0.71, 3.95), chronic = 36.55 μg/kg (25.43, 52.54)], indicating considerable tolerance to the effects of AM2389 on TDMTS performance. The largest dose of AM2389 tested during chronic conditions (56.0 μg/kg) abolished task performance in 2 of 4 subjects.

The left panels of Figure 3 present the effects of rimonabant on mean titrated delay values (upper-left panel) and response rate (lower-left panel) before (1 mg/kg; white diamonds) and during (0.1–1 mg/kg; black diamonds) chronic AM2389 exposure. Rimonabant failed to disrupt mean titrated values either before or during chronic AM2389 exposure (cf. Figure 2, black circle) but did alter rates of responding. Thus, the highest dose of rimonabant, 1.0 mg/kg decreased the mean response rate to approximately 50% of values when studied before the chronic regimen. Lower doses of rimonabant (0.1 and 0.32 mg/kg) also were evaluated during the chronic AM2389 regimen and had differing effects on control values for response rate during the chronic regimen (cf., Figure 2, gray square). Thus, whereas the intermediate dose of rimonabant (0.32 mg/kg) did not appreciably alter those values, the lowest dose of rimonabant (0.1 mg/kg), increased responding sufficiently to recover prechronic response rates, albeit with greater individual variability than observed under other conditions.

The right panels of Figure 3 show the effects of abrupt AM2389 discontinuation on titrated delay (upper-right panel) and response rate (lower-right panel). The white circles and shaded area represent, respectively, the average across the last 5 sessions of the preceding chronic AM2389 condition. As shown by the black circles, mean titrated delay values were reduced during the session on the day following discontinuation, i.e., 27 h after the last injection of AM2389. The disruption in performance increased during the second day following discontinuation, as evident by an approximately ∼60% decrease in titrated delay values. Thereafter, mean titrated delay values gradually recovered across sessions and, by the 10th day following AM2389 discontinuation, began to stabilize at levels of control performance that previously were observed prior to and during chronic exposure (cf. black circle in upper panel of Figure 2). Rates of responding also were disrupted by discontinuation of treatment with AM2389; however, peak disruption was observed 4 days after AM2389 discontinuation. Like titrated delay values, recovery of responding followed and mean response rates approximated previously observed values by the end of the 10-session condition. However, unlike titrated delay values, response rate did not approximate pre-chronic levels (cf. black circle in lower panel of Figure 2) by the end of the 10-session condition.

### Functional magnetic resonance imaging

Independent component analysis identified a prefrontal IC encompassing the mOFC region of interest (see Figure 4; warm colors). This network contained bilateral mOFC, prefrontal cortical regions, and anterior cingulate cortex and overlapped with the mOFC voxel placed for MRS analysis. The dual regression analysis suggested that connectivity decreased from the pre-AM2389 baseline following acute treatment with AM2389 (10 μg/kg; *p* = 0.08). However, chronic exposure to AM2389 (0.32 mg/kg) did not exacerbate this effect but, instead, further increased connectivity within the network (green, *p* < 0.05), especially in mOFC, anterior cingulate, prefrontal cortex, and caudate. However, these effects of chronic AM2389 on functional connectivity were not enduring and were no longer statistically significant 30 days after discontinuation of the chronic regimen (*p* > 0.05).

### Magnetic resonance spectroscopy

Figure 5 shows the ratio of tNAA/tCrw in the mOFC voxel under different experimental conditions (pre-drug baseline, following acute treatment with 10 μg/kg AM2389, after exposure to a total dosage of 0.32 mg/kg AM2389 during chronic treatment, and 30 days following discontinuation of the chronic regimen). ANOVA identified a significant effect of treatment [*F*(3,7) = 13.35; *p* = 0.003] with *post hoc* tests showing that the tNAA/tCrw ratio following acute administration (10 μg/kg) and during the chronic treatment condition (0.32 mg/kg) were significantly decreased relative to baseline ratios (both p’*s* < 0.02). However, this effect was transient as tNAA/tCrw ratio in mOFC 30 days after discontinuation of AM2389 treatment did not differ from baseline levels (*p* > 0.05).

## Discussion

In the present study, acute administration of the CB_1_ full agonist AM2389 produced dose-related disruptions in behavioral and neurochemical measures (tNAA/tCrw ratio) but, notwithstanding a trend toward decreased local frontal connectivity, did not significantly alter connectivity in the mOFC network. During chronic exposure, tolerance developed to the effects of AM2389 on both motoric performance (response rates under the FR20 schedule of food reinforcement) and memorial function (titrated delay in the matching-to-sample procedure; TDMTS) but not to its neurochemical effects in the mOFC. Finally, whereas the administration of the CB_1_ antagonist rimonabant during chronic treatment disrupted motoric performance but not memorial function, discontinuation of the chronic AM2389 regimen disrupted both types of behavior. Overall, the present findings are consistent with the results of previous studies in humans and laboratory animals showing that, first, cannabinoid agonists may produce adverse effects on short-term memory processes (e.g., Abush and Akirav, 2012; Crane et al., 2013; Verrico et al., 2014; Stringfield and Torregrossa, 2021) and second, tolerance develops to such behavioral effects of cannabinoid agonists over the course of chronic exposure (Ramaekers et al., 2011). The magnitude of tolerance observed here was substantial (1.5 log unit rightward shift) and is comparable in magnitude to that reported in previous behavioral studies of cannabinoid tolerance and cross-tolerance in non-human primates (e.g., McMahon, 2011; Desai et al., 2013). It is interesting that tolerance to the effects of the daily dosage of AM2389 appeared more complete on memorial function than on motoric performance. Thus, whereas TDMTS recovered fully during the chronic AM2389 regimen, response rates recovered to only 70% of pre-chronic values and, during dose-response re-determinations, were markedly disrupted by doses of AM2389 that had only moderate effects on titrated delay values. Previous studies have shown that the extent to which acutely administered cannabinoids disrupt performance on cognitive tasks may vary with the difficulty of the task, i.e., cognitive load (Kangas et al., 2016). The present results indicate that tolerance to the disruptive effects of chronically administered cannabinoid agonists also may vary with the class of behavior being measured, i.e., cognitive vs. motoric task performance.

Although the precise defining features and severity of cannabis withdrawal continue to be a subject of inquiry (e.g., Budney and Hughes, 2006; Schlienz et al., 2017), it is now commonly accepted that withdrawal symptoms can emerge following the abrupt discontinuation of cannabis products, especially in high-intake chronic users. This view was codified in the DSM-5 (American Psychiatric Association, 2013) with the inclusion of Cannabis Use Disorder which, for the first time, included diagnostic criteria for cannabis dependence. Moreover, it is widely recognized that such withdrawal discomfort can include pronounced disruptions in cognitive function (Cousijn and van Duijvenvoorde, 2018). The present results showing that discontinuation of AM2389 treatment in the present studies was followed by decrements in short-term memory is consistent with this understanding of cannabis withdrawal. Such effects were most severe on the second day after treatment was discontinued, which is in line with the long duration of action reported for AM2389 (Järbe et al., 2012). The recovery of task performance over the course of approximately 5–10 days also comports well with findings from a previous study of human memory processes in adolescent and young adults showing that 1 month (but as brief as 1 week) of abstinence from cannabis use was associated with improvements in memory task performance (Schuster et al., 2018; see also Scott et al., 2018).

The failure of the CB_1_ receptor antagonist rimonabant to produce withdrawal effects comparable to or, depending on dose, more intense than those observed following discontinuation of the AM2389 regimen was surprising. A defining feature of drug dependence resulting from chronic exposure to other classes of drugs, for example, opioids or benzodiazepines (e.g., Gellert and Holtzman, 1979; Lukas and Griffiths, 1982) is the ability of receptor antagonists to precipitate overt, pronounced signs of withdrawal, and previous studies have shown that the stimulus properties of 24 h abstinence after chronic treatment with Δ^9^-THC or AM2389 overlap with those of rimonabant in non-human primates (Stewart and McMahon, 2010; Kangas et al., 2020). Studies in rodents and non-human primates that are chronically treated with Δ^9^-THC or other CB_1_ agonists have further suggested that rimonabant-precipitated withdrawal can be characterized by overt signs including paw tremor and head shakes (Verberne et al., 1981; Cook et al., 1998; Stewart and McMahon, 2010). However, this remains controversial, as such behavioral disturbances can be produced by rimonabant alone (including psychiatric side effects that led to its withdrawal from market; Nguyen et al., 2019) and are not always apparent in subjects treated with cannabinoids like Δ^9^-THC or AM2389 (Kangas et al., 2020). Interestingly, studies in cannabinoid-treated rodents also have revealed time- and dose-dependent changes in motor activity consequent to rimonabant injection that could serve as a more reliable indicator of cannabinoid withdrawal (Huang et al., 2009; Missig et al., 2022; Paronis et al., 2022). With the latter findings in mind, it is noteworthy that, in the present study, lower doses of rimonabant produced considerable increases in fixed-ratio responding in AM2389-treated monkeys, likely reflecting a mild agitation or motor stimulation. Possibly, assays that are particularly sensitive to such changes in motor activity or assessment of daily patterns of locomotor activity during cannabinoid treatment and discontinuation will provide more consistent demonstrations of antagonist-precipitated cannabinoid withdrawal than are currently available.

Neuroimaging studies in human subjects suggest that use of cannabis during adolescence may lead to abnormalities in brain function, structure, and chemistry in several regions related to cognitive processing (reviewed by Lichenstein et al., 2022; Ma et al., 2022). Studies conducted in adolescent cannabis users have often focused on mOFC, a brain region that undergoes extensive pruning and reorganization during adolescence (Sturman and Moghaddam, 2011) and that is thought to play a role in maintaining drug use in addiction (London et al., 2000; Winstanley, 2007; Schoenbaum and Shaham, 2008). The mOFC also has been linked to performance on cognitive tasks including delayed match-to-sample performance (Lamar et al., 2004). Accordingly, ICA–which revealed a fronto-cortical functional network that primarily encompasses the mOFC, prefrontal cortex, and anterior cingulate—and an MRS voxel in the mOFC were utilized in the present study to probe changes in brain function and chemistry during and after chronic AM2389 treatment. The present results, illustrating greater functional connectivity within a fronto-cortical network during chronic AM2389 treatment, are consistent with human studies showing a relationship between the extent of cannabis use and frontal connectivity (Houck et al., 2013; Orr et al., 2013; Lopez-Larson et al., 2015). For example, increased OFC functional connectivity with other frontal and motor regions (Lopez-Larson et al., 2015) as well as with dorsal striatal regions (Zimmermann et al., 2018) has been demonstrated previously in heavy cannabis users when compared to healthy controls. Importantly, greater OFC connectivity with frontal and subcortical regions also has been associated with greater resistance to behavioral change in high-risk adolescent cannabis users (Feldstein Ewing et al., 2017), consistent with the view that alterations in this network may be a key component of compromised executive control in adolescent cannabis users. In the present study, changes in RSFC within this network could be related to changes in performance on the TDMTS task. For example, RSFC within the fronto-cortical network showed a non-significant trend for decreased connectivity following acute treatment with a behaviorally disruptive dose of AM2389 (10 ug/kg). In contrast, RSFC increased within the frontal network following a regimen of daily AM2389 administration that induced tolerance to its performance–disrupting effects. It is tempting to speculate that increased connectivity among frontal regions during chronic AM2389 treatment represents a recruitment of brain regions that helped to overcome the disruptive behavioral effects of AM2389 that were evident after acute administration. These effects were not persistent, however, as functional connectivity following treatment discontinuation was able to recover to pre-treatment levels.

The combined use of fMRI and MRS in the same subjects provided the opportunity to gain insights into the neurochemical mechanisms that may contribute to drug-induced changes in functional connectivity (Kohut and Kaufman, 2021). Here, we showed that the tNAA/tCrw ratio in mOFC decreased in a dose-dependent manner during AM2389 treatment but returned to baseline levels following discontinuation. We focused on NAA levels because they are thought to be a marker of neuronal health and integrity and have been shown to be altered in substance use disorders (Magalhaes, 2005), including cannabis use disorder. For example, increased striatal NAA has been positively correlated to subjective reports of “high” following acute THC administration to occasional cannabis users (Mason et al., 2021). On the other hand, decreased NAA concentration has been reported in frontal regions such as anterior cingulate cortex (Prescot et al., 2011) and dorsolateral prefrontal cortex (Hermann et al., 2007) in chronic cannabis users suggesting that the effects of cannabis on brain NAA may be region-dependent or change with chronic exposure. Further, Sung et al. (2013) showed decreased frontal NAA levels in adolescents that use methamphetamine + marijuana compared to levels in methamphetamine only users or healthy controls; NAA ratios were also inversely related to lifetime and duration of marijuana use. The relationship between neurochemical environment in a given brain region and its functional connectivity is likely to be complex and influenced by multiple factors, Lord et al. (2017) described a quadratic relationship between NAA concentration and functional connectivity in posterior cingulate cortex of healthy volunteers in which functional connectivity and NAA decreased linearly before increasing again. While a causative influence of NAA on functional connectivity (or vice versa) cannot be proven from data in the present study, it is noteworthy that the effects of AM2389 administration generally followed this same pattern. Further studies that target other metabolites in frontal regions may shed greater light on how cannabinoids alter the relationship between neurochemical indices and functional connectivity.

There are several caveats and limitations of these studies that deserve mention. First, all adolescent rhesus macaques examined were female. As greater CB_1_ density in several brain regions has been consistently reported in males than females in laboratory animal studies (e.g., Rubino et al., 2008; Reich et al., 2009; Burston et al., 2010; Mateos et al., 2011), it will be important for future studies to determine whether the present findings can be replicated in adolescent male monkeys. Second, relating hormonal phases to behavioral and neuroimaging endpoints was beyond the scope of the present studies and, therefore, not systematically assessed. Interestingly, although these subjects had not begun menses prior to the initiation of these studies, first menses and subsequent cycling were observed in all subjects at various points during these studies, confirming that these experiments indeed were occurring during the intended developmental stage. Also, magnetic resonance spectroscopy conducted at 3T field strength was not sensitive enough in non-human primates to distinguish resonance peaks for other neurochemicals of interest such as glutamate, y-aminobutyric acid (GABA), and glutamine. Future studies using other acquisition techniques or ultra-high field scanners to enhance the signal of such metabolites will help further characterize the neurochemical environment in the mOFC and related brain regions during chronic drug administration (Ma et al., 2022). Next, although accuracy in the TDMTS task was adversely affected by acute AM2389 administration as well as discontinuation of chronic treatment, it was noteworthy that all rewards earned by subjects were indeed consumed. Despite this, a decrease in motivation for the milk reinforcer cannot be ruled out as the behavioral mechanism of observed task disruption and, as such, the effects of chronic AM2389 treatment and discontinuation upon TDMTS performance maintained by a non-food consequence will be necessary to examine this possibility. Finally, it is important to note that inasmuch as these studies employed a within-subject behavioral and neuroimaging design during pre-drug exposure, an untreated control group was not included during or following chronic exposure. While a drug-free control group would have allowed for interesting comparisons of behavioral and neuroimaging endpoints during these phases of this research, the longitudinal design of the present study nonetheless clearly reveals modulation of both cognitive and MRS/fMRI metrics during acute and chronic cannabinoid exposure and, as well, their normalization following its discontinuation.

## Data availability statement

The raw data supporting the conclusions of this article will be made available by the authors, without undue reservation.

## Ethics statement

The animal study was reviewed and approved by McLean Hospital Animal Care and Use Committee.

## Author contributions

SK, JB, BF, JJ, and BK participated in research design. SK, CZ, JJ, and BK conducted the experiments. SJ, SN, and AM contributed the new reagents or analytic tools. DM, LC, BF, SK, and BK performed the data analysis. SK, LC, DM, JB, and BK contributed the writing of the manuscript. All authors contributed to the article and approved the submitted version.

## Footnotes

1
http://www.jmrui.eu/

2
http://www.nmr.mgh.harvard.edu/~jbm/jip/

## References

[B1] Abush H. Akirav I. (2012). Short- and long-term cognitive effects of chronic cannabinoids administration in late-adolescence rats. *PLoS One* 7:e31731. 10.1371/journal.pone.0031731 22348124 PMC3278466

[B2] American Psychiatric Association (2013). *Desk reference to the diagnostic criteria from DSM-5*. Washington, DC: American Psychiatric Association.

[B3] Bava S. Jacobus J. Mahmood O. Yang T. T. Tapert S. F. (2010). Neurocognitive correlates of white matter quality in adolescent substance users. *Brain Cogn.* 72 347–354. 10.1016/j.bandc.2009.10.012 19932550 PMC2832074

[B4] Becker J. B. Hu M. (2008). Sex differences in drug abuse. *Front. Neuroendocrinol.* 29 36–47. 10.1016/j.yfrne.2007.07.003 17904621 PMC2235192

[B5] Bevington P. R. Robinson D. K. Blair J. M. Mallinckrodt A. J. McKay S. (1993). Data Reduction and Error Analysis for the Physical Sciences. *Comput. Phys.* 7:415.

[B6] Budney A. J. Hughes J. R. (2006). The cannabis withdrawal syndrome. *Curr. Opin. Psychiatry* 19 233–238. 10.1097/01.yco.0000218592.00689.e5 16612207

[B7] Burston J. J. Wiley J. L. Craig A. A. Selley D. E. Sim-Selley L. J. (2010). Regional enhancement of cannabinoid CB_1_ receptor desensitization in female adolescent rats following repeated Delta-tetrahydrocannabinol exposure. *Br. J. Pharmacol.* 161 103–112. 10.1111/j.1476-5381.2010.00870.x 20718743 PMC2962820

[B8] Cadoni C. Pisanu A. Solinas M. Acquas E. Di Chiara G. (2001). Behavioural sensitization after repeated exposure to Delta 9-tetrahydrocannabinol and cross-sensitization with morphine. *Psychopharmacology (Berl)* 158, 259–66. 10.1007/s002130100875 11713615

[B9] Camchong J. Lim K. O. Kumra S. (2017). Adverse effects of cannabis on adolescent brain development: A longitudinal study. *Cereb. Cortex* 27 1922–1930. 26912785 10.1093/cercor/bhw015 PMC5963818

[B10] Cha Y. M. Jones K. H. Kuhn C. M. Wilson W. A. Swartzwelder H. S. (2007). Sex differences in the effects of delta9-tetrahydrocannabinol on spatial learning in adolescent and adult rats. *Behav. Pharmacol.* 18 563–569. 17762524 10.1097/FBP.0b013e3282ee7b7e

[B11] Cheetham A. Allen N. B. Whittle S. Simmons J. G. Yucel M. Lubman D. I. (2012). Orbitofrontal volumes in early adolescence predict initiation of cannabis use: A 4-year longitudinal and prospective study. *Biol. Psychiatry* 71 684–692. 10.1016/j.biopsych.2011.10.029 22129756

[B12] Cheng H. Skosnik P. D. Pruce B. J. Brumbaugh M. S. Vollmer J. M. Fridberg D. J. (2014). Resting state functional magnetic resonance imaging reveals distinct brain activity in heavy cannabis users: A multi-voxel pattern analysis. *J. Psychopharmacol.* 28 1030–1040. 10.1177/0269881114550354 25237118 PMC4427512

[B13] Churchwell J. C. Lopez-Larson M. Yurgelun-Todd D. A. (2010). Altered Frontal Cortical Volume and Decision Making in Adolescent Cannabis Users. *Front. Psychol.* 1:225. 10.3389/fpsyg.2010.00225 21833280 PMC3153830

[B14] Cook S. A. Lowe J. A. Martin B. R. (1998). CB1 receptor antagonist precipitates withdrawal in mice exposed to Delta9-tetrahydrocannabinol. *J. Pharmacol. Exp. Ther.* 285 1150–1156. 9618417

[B15] Cousijn J. van Duijvenvoorde A. C. K. (2018). Cognitive and Mental Health Predictors of Withdrawal Severity During an Active Attempt to Cut Down Cannabis Use. *Front. Psychiatry* 11:301. 10.3389/fpsyt.2018.00301 30050473 PMC6050370

[B16] Crane N. A. Schuster R. M. Gonzalez R. (2013). Preliminary evidence for a sex-specific relationship between amount of cannabis use and neurocognitive performance in young adult cannabis users. *J. Int. Neuropsychol. Soc.* 19 1009–1015. 10.1017/S135561771300088X 23962414 PMC3895398

[B17] Crean R. D. Crane N. A. Mason B. J. (2011). An evidence based review of acute and long-term effects of cannabis use on executive cognitive functions. *J. Addict. Med.* 5 1–8. 21321675 10.1097/ADM.0b013e31820c23fa PMC3037578

[B18] Delevich K. Klinger M. Okada N. J. Wilbrecht L. (2021). Coming of age in the frontal cortex: The role of puberty in cortical maturation. *Semin. Cell Dev. Biol.* 118 64–72. 10.1016/j.semcdb.2021.04.021 33985902 PMC12018213

[B19] Desai R. I. Thakur G. A. Vemuri V. K. Bajaj S. Makriyannis A. Bergman J. (2013). Analysis of tolerance and behavioral/physical dependence during chronic CB1 agonist treatment: Effects of CB1 agonists, antagonists, and noncannabinoid drugs. *J. Pharmacol. Exp. Ther.* 344 319–328. 10.1124/jpet.112.198374 23197773 PMC3558821

[B20] Egerton A. Allison C. Brett R. R. Pratt J. A. (2006). Cannabinoids and prefrontal cortical function: Insights from preclinical studies. *Neurosci. Biobehav. Rev.* 30 680–695. 16574226 10.1016/j.neubiorev.2005.12.002

[B21] Feldstein Ewing S. W. Chung T. Caouette J. D. Ketcherside A. Hudson K. A. Filbey F. M. (2017). Orbitofrontal cortex connectivity as a mechanism of adolescent behavior change. *Neuroimage* 151 14–23. 10.1016/j.neuroimage.2016.12.076 28039093 PMC5420345

[B22] Fontes M. A. Bolla K. I. Cunha P. J. Almeida P. P. Jungerman F. Laranjeira R. R. (2011). Cannabis use before age 15 and subsequent executive functioning. *Br. J. Psychiatry* 198 442–447. 10.1192/bjp.bp.110.077479 21628706

[B23] Gellert V. F. Holtzman S. G. (1979). Discriminative stimulus effects of naltrexone in the morphine-dependent rat. *J. Pharmacol. Exp. Ther.* 211 596–605. 574545

[B24] Gruber S. A. Yurgelun-Todd D. A. (2005). Neuroimaging of marijuana smokers during inhibitory processing: A pilot investigation. *Brain Res. Cogn. Brain Res* 23 107–118. 10.1016/j.cogbrainres.2005.02.016 15795138

[B25] Gruber S. A. Sagar K. A. Dahlgren M. K. Racine M. Lukas S. E. (2012). Age of onset of marijuana use and executive function. *Psychol. Addict. Behav.* 26 496–506. 10.1037/a0026269 22103843 PMC3345171

[B26] Hanson K. L. Medina K. L. Padula C. B. Tapert S. F. Brown S. A. (2011). How does adolescent alcohol and drug use affect neuropsychological functioning in young adulthood: 10-year outcomes. *J. Child Adolesc. Subst. Abuse* 20 135–154. 10.1080/1067828X.2011.555272 21532924 PMC3083020

[B27] Hermann D. Sartorius A. Welzel H. Walter S. Skopp G. Ende G. (2007). Dorsolateral prefrontal cortex N-acetylaspartate/total creatine (NAA/tCr) loss in male recreational cannabis users. *Biol. Psychiatry.* 61 1281–1289. 10.1016/j.biopsych.2006.08.027 17239356

[B28] Higuera-Matas A. Botreau F. Miguéns M. Del Olmo N. Borcel E. Pérez-Alvarez L. (2009). Chronic periadolescent cannabinoid treatment enhances adult hippocampal PSA-NCAM expression in male Wistar rats but only has marginal effects on anxiety, learning and memory. *Pharmacol. Biochem. Behav.* 93 482–490. 10.1016/j.pbb.2009.06.013 19576923

[B29] Houck J. M. Bryan A. D. Feldstein Ewing S. W. (2013). Functional connectivity and cannabis use in high-risk adolescents. *Am. J. Drug. Alcohol. Abuse* 39 414–423. 10.3109/00952990.2013.837914 24200211 PMC4070738

[B30] Huang P. Liu-Chen L. Y. Unterwald E. M. Cowan A. (2009). Hyperlocomotion and paw tremors are two highly quantifiable signs of SR141716-precipitated withdrawal from delta9-tetrahydrocannabinol in C57BL/6 mice. *Neurosci. Lett.* 465 66–70. 10.1016/j.neulet.2009.08.073 19733208 PMC11192173

[B31] Huettel S. A. (2012). Event-related fMRI in cognition. *NeuroImage* 62 1152–1156. 21963919 10.1016/j.neuroimage.2011.08.113 PMC3277683

[B32] Hurd R. Sailasuta N. Srinivasan R. Vigneron D. B. Pelletier D. Nelson S. J. (2004). Measurement of brain glutamate using TE-averaged PRESS at 3T. *Magn. Reson. Med.* 51 435–440. 10.1002/mrm.20007 15004781

[B33] Jacobus J. Bava S. Cohen-Zion M. Mahmood O. Tapert S. F. (2009). Functional consequences of marijuana use in adolescents. *Pharmacol. Biochem. Behav.* 92 559–565. 19348837 10.1016/j.pbb.2009.04.001 PMC2697065

[B34] Järbe T. U. Tai S. LeMay B. J. Nikas S. P. Shukla V. G. Zvonok A. (2012). AM2389, a high-affinity, in vivo potent CB1-receptor-selective cannabinergic ligand as evidenced by drug discrimination in rats and hypothermia testing in mice. *Psychopharmacology* 220 417–426. 10.1007/s00213-011-2491-1 21989802 PMC3291515

[B35] Jensen J. E. Licata S. C. Ongür D. Friedman S. D. Prescot A. P. Henry M. E. (2009). Quantification of J-resolved proton spectra in two-dimensions with LCModel using GAMMA-simulated basis sets at 4 Tesla. *NMR Biomed.* 22 762–769. 10.1002/nbm.1390 19388001

[B36] Kangas B. D. Bergman J. (2012). A novel touch-sensitive apparatus for behavioral studies in unrestrained squirrel monkeys. *J. Neurosci. Methods* 209, 331–336. 10.1016/j.jneumeth.2012.06.028 22790109 PMC3429786

[B37] Kangas B. D. Bergman J. (2017). Touchscreen technology in the study of cognition-related behavior. *Behav. Pharmacol.* 28 623–629. 10.1097/FBP.0000000000000356 29064843 PMC5687822

[B38] Kangas B. D. Branch M. N. (2008). Empirical validation of a procedure to correct position and stimulus biases in matching-to-sample. *J. Exp. Anal. Behav.* 90 103–112. 10.1901/jeab.2008.90-103 18683615 PMC2441575

[B39] Kangas B. D. Leonard M. Z. Shukla V. G. Alapafuja S. O. Nikas S. P. Makriyannis A. (2016). Comparisons of Δ9-Tetrahydrocannabinol and Anandamide on a Battery of Cognition-Related Behavior in Nonhuman Primates. *J. Pharmacol. Exp. Ther.* 357 125–133. 10.1124/jpet.115.228189 26826191 PMC4809315

[B40] Kangas B. D. Vaidya M. Branch M. N. (2010). Titrating-delay matching-to-sample in the pigeon. *J. Exp. Anal. Behav.* 94 69–81. 10.1901/jeab.2010.94-69 21279163 PMC2893618

[B41] Kangas B. D. Zakarian A. S. Vemuri K. Alapafuja S. O. Jiang S. Nikas S. P. (2020). Cannabinoid Antagonist Drug Discrimination in Nonhuman Primates. *J. Pharmacol. Exp. Ther.* 372 119–127. 10.1124/jpet.119.261818 31641018 PMC6927407

[B42] Kohut S. J. Kaufman M. J. (2021). Magnetic resonance spectroscopy studies of substance use disorders: Current landscape and potential future directions. *Pharmacol. Biochem. Behav.* 200:173090. 10.1016/j.pbb.2020.173090 33333132 PMC8050617

[B43] Lamar M. Yousem D. M. Resnick S. M. (2004). Age differences in orbitofrontal activation: An fMRI investigation of delayed match and nonmatch to sample. *Neuroimage* 21 1368–1376. 10.1016/j.neuroimage.2003.11.018 15050562

[B44] Lichenstein S. D. Manco N. Cope L. M. Egbo L. Garrison K. A. Hardee J. (2022). Systematic review of structural and functional neuroimaging studies of cannabis use in adolescence and emerging adulthood: Evidence from 90 studies and 9441 participants. *Neuropsychopharmacology* 47 1000–1028. 10.1038/s41386-021-01226-9 34839363 PMC8938408

[B45] London E. D. Ernst M. Grant S. Bonson K. Weinstein A. (2000). Orbitofrontal cortex and human drug abuse: Functional imaging. *Cereb. Cortex* 10 334–342. 10.1093/cercor/10.3.334 10731228

[B46] Lopez-Larson M. P. Rogowska J. Yurgelun-Todd D. (2015). Aberrant orbitofrontal connectivity in marijuana smoking adolescents. *Dev. Cogn. Neurosci.* 16 54–62. 10.1016/j.dcn.2015.08.002 26296778 PMC4691408

[B47] Lord A. R. Li M. Demenescu L. R. van den Meer J. Borchardt V. Krause A. L. (2017). Richness in Functional Connectivity Depends on the Neuronal Integrity within the Posterior Cingulate Cortex. *Front. Neurosci.* 11:184. 10.3389/fnins.2017.00184 28439224 PMC5384321

[B48] Lorenzetti V. Solowij N. Yücel M. (2016). The Role of Cannabinoids in Neuroanatomic Alterations in Cannabis Users. *Biol. Psychiatry* 79 e17–e31. 26858212 10.1016/j.biopsych.2015.11.013

[B49] Lubman D. I. Cheetham A. Yücel M. (2015). Cannabis and adolescent brain development. *Pharmacol. Ther.* 148 1–16. 25460036 10.1016/j.pharmthera.2014.11.009

[B50] Lukas S. E. Griffiths R. R. (1982). Precipitated withdrawal by a benzodiazepine receptor antagonist (Ro 15-1788) after 7 days of diazepam. *Science* 217 1161–1163. 10.1126/science.6287579 6287579

[B51] Ma J. Lyoo I. K. Renshaw P. F. Yurgelun-Todd D. A. (2022). Effect of cannabinoids on brain metabolites: A review of animal and human studies. *Exp. Clin. Psychopharmacol.* [Epub ahead of print]. 10.1037/pha0000553 35201831

[B52] Magalhaes A. C. (2005). Functional Magnetic Resonance and Spectroscopy in Drug and Substance Abuse. *Top. Magn. Reson. Imaging* 16 247–251. 10.1097/01.rmr.0000194048.43739.d4 16340649

[B53] Mason N. L. Theunissen E. L. Hutten N. R. P. W. Tse D. H. Y. Toennes S. W. Jansen J. F. A. (2021). Reduced responsiveness of the reward system is associated with tolerance to cannabis impairment in chronic users. *Addict. Biol.* 26:e12870. 10.1111/adb.12870 31865628 PMC7757162

[B54] Mateos B. Borcel E. Loriga R. Luesu W. Bini V. Llorente R. (2011). Adolescent exposure to nicotine and/or the cannabinoid agonist CP 55,940 induces gender-dependent long-lasting memory impairments and changes in brain nicotinic and CB(1) cannabinoid receptors. *J. Psychopharmacol.* 25 1676–1690. 10.1177/0269881110370503 20562169

[B55] McMahon L. R. (2011). Chronic Δ^9^-tetrahydrocannabinol treatment in rhesus monkeys: Differential tolerance and cross-tolerance among cannabinoids. *Br. J. Pharmacol.* 162 1060–1073. 10.1111/j.1476-5381.2010.01116.x 21091643 PMC3051379

[B56] Mintzopoulos D. Gillis T. E. Robertson H. R. Dalia T. Feng G. Rauch S. L. (2016). Striatal magnetic resonance spectroscopy abnormalities in young adult SAPAP3 knockout mice. *Biol. Psychiatry Cogn. Neurosci. Neuroimaging* 1 39–48. 10.1016/j.bpsc.2015.10.001 26858992 PMC4742338

[B57] Missig G. Mehta N. Robbins J. O. Good C. H. Iliopoulos-Tsoutsouvas C. Makriyannis A. (2022). Altered sleep during spontaneous cannabinoid withdrawal in male mice. *Behav. Pharmacol.* 33 195–205. 10.1097/FBP.0000000000000674 35288510 PMC8928162

[B58] Moss H. B. Chen C. M. Yi H.-Y. (2014). Early adolescent patterns of alcohol, cigarettes, and marijuana polysubstance use and young adult substance use outcomes in a nationally representative sample. *Drug Alcohol Depend* 136 51–62. 10.1016/j.drugalcdep.2013.12.011 24434016

[B59] National Research Council (2010). *Guide for the care and use of laboratory animals*. Washington, DC: National Academies Press.

[B60] Nguyen T. Thomas B. Zhang Y. (2019). Overcoming the psychiatric side effects of cannabinoid CB1 receptor antagonists: Current approaches for therapeutic development. *Curr. Top. Med. Chem.* 19 1418–1435. 10.2174/1568026619666190708164841 31284863 PMC6771026

[B61] Nikas S. P. Alapafuja S. O. Papanastasiou I. Paronis C. A. Shukla V. G. Papahatjis D. P. (2010). Novel 1’,1’-chain substituted hexahydrocannabinols: 9β-hydroxy-3-(1-hexyl-cyclobut-1-yl)-hexahydrocannabinol (AM2389) a highly potent cannabinoid receptor 1 (CB1) agonist. *J. Med. Chem.* 53 6996–7010. 10.1021/jm100641g 20925434 PMC3650853

[B62] O’Shea M. McGregor I. S. Mallet P. E. (2006). Repeated cannabinoid exposure during perinatal, adolescent or early adult ages produces similar longlasting deficits in object recognition and reduced social interaction in rats. *J. Psychopharmacol.* 20 611–621. 10.1177/0269881106065188 16714325

[B63] O’Shea M. Singh M. E. McGregor I. S. Mallet P. E. (2004). Chronic cannabinoid exposure produces lasting memory impairment and increased anxiety in adolescent but not adult rats. *J. Psychopharmacol.* 18 502–508. 10.1177/026988110401800407 15582916

[B64] Orr C. Morioka R. Behan B. Datwani S. Doucet M. Ivanovic J. (2013). Altered resting-state connectivity in adolescent cannabis users. *Am. J. Drug Alcohol Abuse* 39 372–381. 10.3109/00952990.2013.848213 24200207

[B65] Padula C. B. Schweinsburg A. D. Tapert S. F. (2007). Spatial working memory performance and fMRI activation interactions in abstinent adolescent marijuana users. *Psychol. Addict. Behav.* 21 478–487. 10.1037/0893-164X.21.4.478 18072830 PMC2373252

[B66] Paronis C. A. Iliopoulos-Tsoutsouvas C. Papanastasiou I. Makriyannis A. Bergman J. Nikas S. P. (2022). Evidence for spontaneous cannabinoid withdrawal in mice. *Behav. Pharmacol.* 33 184–194. 10.1097/FBP.0000000000000665 35288509 PMC8924453

[B67] Paronis C. A. Nikas S. P. Shukla V. G. Makriyannis A. (2012). Δ(9)-Tetrahydrocannabinol acts as a partial agonist/antagonist in mice. *Behav. Pharmacol.* 23 802–805. 10.1097/FBP.0b013e32835a7c4d 23075707 PMC3697741

[B68] Peters K. Z. Zlebnik N. E. Cheer J. F. (2022). Cannabis exposure during adolescence: A uniquely sensitive period for neurobiological effects. *Int. Rev. Neurobiol.* 161 95–120. 10.1016/bs.irn.2021.07.002 34801175

[B69] Posse S. Otazo R. Caprihan A. Bustillo J. Chen H. Henry P. G. (2007). Proton echo-planar spectroscopic imaging of J-coupled resonances in human brain at 3 and 4 Tesla. *Magn. Reson. Med.* 58 236–244. 10.1002/mrm.21287 17610279

[B70] Prescot A. P. Locatelli A. E. Renshaw P. F. Yurgelun-Todd D. A. (2011). Neurochemical alterations in adolescent chronic marijuana smokers: A proton MRS study. *Neuroimage* 57 69–75. 10.1016/j.neuroimage.2011.02.044 21349338 PMC3101285

[B71] Quinn H. R. Matsumoto I. Callaghan P. D. Long L. E. Arnold J. C. Gunasekaran N. (2008). Adolescent rats find repeated delta(9)-THC less aversive than adult rats but display greater residual cognitive deficits and changes in hippocampal protein expression following exposure. *Neuropsychopharmacology* 33 1113–1126. 10.1038/sj.npp.1301475 17581536

[B72] Ramaekers J. G. Theunissen E. L. de Brouwer M. Toennes S. W. Moeller M. R. Kauert G. (2011). Tolerance and cross-tolerance to neurocognitive effects of THC and alcohol in heavy cannabis users. *Psychopharmacology* 214 391–401. 10.1007/s00213-010-2042-1 21049267 PMC3045517

[B73] Reich C. G. Taylor M. E. McCarthy M. M. (2009). Differential effects of chronic unpredictable stress on hippocampal CB1 receptors in male and female rats. *Behav. Brain Res.* 203 264–269. 10.1016/j.bbr.2009.05.013 19460405 PMC2747651

[B74] Rohlfing T. Kroenke C. D. Sullivan E. V. Dubach M. F. Bowden D. M. Grant K. A. (2012). The INIA19 Template and NeuroMaps Atlas for Primate Brain Image Parcellation and Spatial Normalization. *Front. Neuroinform.* 6:27. 10.3389/fninf.2012.00027 23230398 PMC3515865

[B75] Rubino T. Vigano’ D. Realini N. Guidali C. Braida D. Capurro V. (2008). Chronic delta 9-tetrahydrocannabinol during adolescence provokes sex-dependent changes in the emotional profile in adult rats: Behavioral and biochemical correlates. *Neuropsychopharmacology* 33 2760–2771. 10.1038/sj.npp.1301664 18172430

[B76] Sagar K. A. Dahlgren M. K. Gönenç A. Racine M. T. Dreman M. W. Gruber S. A. (2015). The impact of initiation: Early onset marijuana smokers demonstrate altered Stroop performance and brain activation. *Dev. Cogn. Neurosci.* 16 84–92. 10.1016/j.dcn.2015.03.003 25936584 PMC4596753

[B77] Sager K. A. Gruber S. A. (2019). Interactions between recreational cannabis use and cogntive function: Lessons from functional magnetic resonance imaging. *Ann. N.Y. Acad. Sci.* 1451 42–70. 10.1111/nyas.13990 30426517 PMC6513728

[B78] Saleem K. S. Logothetis N. K. (2012). *A Combined MRI and Histology Atlas of the Rhesus Monkey Brain in Stereotaxic Coordinates.* Cambridge, MA: Academic Press.

[B79] Schlienz N. J. Budney A. J. Lee D. C. Vandrey R. (2017). Cannabis withdrawal: A review of neurobiological mechanisms and sex differences. *Curr. Addict. Rep.* 4 75–81. 10.1007/s40429-017-0143-1 29057200 PMC5648025

[B80] Schoenbaum G. Shaham Y. (2008). The role of orbitofrontal cortex in drug addiction: A review of preclinical studies. *Biol. Psychiatry* 63 256–262. 10.1016/j.biopsych.2007.06.003 17719014 PMC2246020

[B81] Schuster R. M. Gilman J. Schoenfeld D. Evenden J. Hareli M. Ulysse C. (2018). One Month of Cannabis Abstinence in Adolescents and Young Adults Is Associated With Improved Memory. *J. Clin. Psychiatry* 79:17m11977. 10.4088/JCP.17m11977 30408351 PMC6587572

[B82] Schweinsburg A. D. Nagel B. J. Schweinsburg B. C. Park A. Theilmann R. J. Tapert S. F. (2008). Abstinent adolescent marijuana users show altered fMRI response during spatial working memory. *Psychiatry Res.* 163 40–51. 18356027 10.1016/j.pscychresns.2007.04.018 PMC2832586

[B83] Scott J. C. Slomiak S. T. Jones J. D. Rosen A. F. G. Moore T. M. Gur R. C. (2018). Association of cannabis with cognitive functioning in adolescents and young adults: A systematic review and meta-analysis. *JAMA Psychiatry* 75 585–595. 10.1001/jamapsychiatry.2018.0335 29710074 PMC6137521

[B84] Smith S. M. Nichols T. E. (2009). Threshold-free cluster enhancement: Addressing problems of smoothing, threshold dependence and localisation in cluster inference. *Neuroimage* 44 83–98. 10.1016/j.neuroimage.2008.03.061 18501637

[B85] Smith S. M. Fox P. T. Miller K. L. Beckmann C. F. (2009). Correspondence of the brain’s functional architecture during activation and rest. *Proc. Natl. Acad. Sci. U.S.A.* 106 13040–13045. 19620724 10.1073/pnas.0905267106 PMC2722273

[B86] Soher B. J. Young K. Bernstein A. Aygula Z. Maudsley A. A. (2007). spectral simulation for in vivo MRS applications. *J. Magn. Reson.* 185 291–299. 17257868 10.1016/j.jmr.2007.01.005 PMC1940040

[B87] Stewart J. L. McMahon L. R. (2010). Rimonabant-induced Delta9-tetrahydrocannabinol withdrawal in rhesus monkeys: Discriminative stimulus effects and other withdrawal signs. *J. Pharmacol. Exp. Ther.* 334 347–356. 10.1124/jpet.110.168435 20375197 PMC2912042

[B88] Stringfield S. J. Torregrossa M. M. (2021). Intravenous self-administration of delta-9-THC in adolescent rats produces long-lasting alterations in behavior and receptor protein expression. *Psychopharmacology* 238 305–319. 10.1007/s00213-020-05684-9 33111197 PMC7796919

[B89] Sturman D. A. Moghaddam B. (2011). The neurobiology of adolescence: Changes in brain architecture, functional dynamics, and behavioral tendencies. *Neurosci. Biobehav. Rev.* 35 1704–1712. 10.1016/j.neubiorev.2011.04.003 21527288 PMC3222328

[B90] Sung Y. H. Carey P. D. Stein D. J. Ferrett H. L. Spottiswoode B. S. Renshaw P. F. (2013). Decreased frontal N-acetylaspartate levels in adolescents concurrently using both methamphetamine and marijuana. *Behav. Brain Res.* 246 154–161. 10.1016/j.bbr.2013.02.028 23466689 PMC3668971

[B91] Tait R. J. Mackinnon A. Christensen H. (2011). Cannabis use and cognitive function: 8-year trajectory in a young adult cohort. *Addiction* 106 2195–2203. 10.1111/j.1360-0443.2011.03574.x 21749524

[B92] Tapert S. F. Schweinsburg A. D. Drummond S. P. Paulus M. P. Brown S. A. Yang T. T. (2007). Functional MRI of inhibitory processing in abstinent adolescent marijuana users. *Psychopharmacology* 194 173–183. 17558500 10.1007/s00213-007-0823-y PMC2269705

[B93] Verberne A. J. Taylor D. A. Fennessy M. R. (1981). Attenuation of delta 9-tetrahydrocannabinol-induced withdrawal-like behaviour by delta 9-tetrahydrocannabinol. *Psychopharmacology* 73 97–98. 10.1007/BF00431112 6262850

[B94] Verrico C. D. Gu H. Peterson M. L. Sampson A. R. Lewis D. A. (2014). Repeated Δ9-tetrahydrocannabinol exposure in adolescent monkeys: Persistent effects selective for spatial working memory. *Am. J. Psychiatry* 171 416–425. 10.1176/appi.ajp.2013.13030335 24577206 PMC4012614

[B95] Verrico C. D. Liu S. Bitler E. J. (2012). Delay- and Dose-Dependent Effects of Δ9-Tetrahydrocannabinol Administration on Spatial and Object Working Memory Tasks in Adolescent Rhesus Monkeys. *Neuropsychopharmacology* 37 1357–1366. 10.1038/npp.2011.321 22218091 PMC3327841

[B96] Weerts E. M. Fantegrossi W. E. Goodwin A. K. (2007). The value of nonhuman primates in drug abuse research. *Exp. Clin. Psychopharmacol.* 15 309–327. 17696678 10.1037/1064-1297.15.4.309

[B97] Winstanley C. A. (2007). The orbitofrontal cortex, impulsivity, and addiction: Probing orbitofrontal dysfunction at the neural, neurochemical, and molecular level. *Ann. N.Y. Acad. Sci.* 1121 639–655. 10.1196/annals.1401.024 17846162

[B98] Woitowich N. C. Beery A. Woodruff T. A. (2020). 10-year follow-up study of sex inclusion in the biological sciences. *elife* 9:e56344. 32513386 10.7554/eLife.56344 PMC7282816

[B99] Wrege J. Schmidt A. Walter A. Smieskova R. Bendfeldt K. Radue E. W. (2014). Effects of cannabis on impulsivity: A systematic review of neuroimaging findings. *Curr. Pharm. Des* 20 2126–2137. 10.2174/13816128113199990428 23829358 PMC4052819

[B100] Zamberletti E. Beggiato S. Steardo L. Prini P. Antonelli T. Ferraro L. (2014). Alterations of prefrontal cortex GABAergic transmission in the complex psychotic-like phenotype induced by adolescent delta-9-tetrahydrocannabinol exposure in rats. *Neurobiol. Dis.* 63 35–47. 10.1016/j.nbd.2013.10.028 24200867

[B101] Zanettini C. Panlilio L. V. Alicki M. Goldberg S. R. Haller J. Yasar S. (2011). Effects of endocannabinoid system modulation on cognitive and emotional behavior. *Front. Behav. Neurosci.* 5:57. 10.3389/fnbeh.2011.00057 21949506 PMC3171696

[B102] Zimmermann K. Yao S. Heinz M. Zhou F. Dau W. Banger M. (2018). Altered orbitofrontal activity and dorsal striatal connectivity during emotion processing in dependent marijuana users after 28 days of abstinence. *Psychopharmacology* 235 849–859. 10.1007/s00213-017-4803-6 29197984

